# Diversity and Long-Term Dynamics of Human Blood Anelloviruses

**DOI:** 10.1128/jvi.00109-22

**Published:** 2022-05-16

**Authors:** Joanna Kaczorowska, Martin Deijs, Michelle Klein, Margreet Bakker, Maarten F. Jebbink, Mila Sparreboom, Cormac M. Kinsella, Anne L. Timmerman, Lia van der Hoek

**Affiliations:** a Amsterdam UMC, University of Amsterdam, Department of Medical Microbiology and Infection Prevention, Laboratory of Experimental Virology, Amsterdam, The Netherlands; b Amsterdam Institute for Infection and Immunity, Amsterdam, The Netherlands; Cornell University

**Keywords:** anellome, anellovirus, blood virome, chronic viral infection, torque teno virus, virome

## Abstract

Anelloviruses (AVs) are commensal members of the human blood virome. Even though it was estimated that over 90% of the human population carries AVs, the dynamics of the AV virome (“anellome”) are unknown. We investigated the dynamics of blood anellomes in two healthy people followed up for more than 30 years. Both subjects were positive for AVs in the majority of samples. *Alphatorquevirus* (torque teno virus [TTV]) was the most common genus in both subjects, followed by *Betatorquevirus* (torque teno minivirus [TTMV]) and *Gammatorquevirus* (torque teno midivirus [TTMDV]). Almost five times more lineages were found in subject 1 than in subject 2, and the anellomes differed phylogenetically. Both anellomes remained compositionally stable, and 9 out of 64 AV lineages were detected in over half of the time points. We confirmed the long-term and short-term persistence of 13 lineages by specific quantitative PCR (qPCR). AV lineages were detected in blood for over 30 years. Noticeable differences in anellome richness were found between the tested subjects, but both anellomes remained compositionally stable over time. These findings demonstrate that the human blood anellome is personal and that AV infection is chronic and potentially commensal.

**IMPORTANCE** Knowledge of the persistence of AVs in humans is crucial to our understanding of the nature of AV infection (chronic or acute) and the role of AV in the host. We therefore investigated the dynamics of anellovirus infection in two healthy people followed up for 30 years. Our findings suggest that the human blood anellovirus virome (anellome) remains stable and personal for decades.

## INTRODUCTION

The blood of nonseptic humans was considered sterile until next-generation sequencing (NGS) of blood samples revealed the presence of viruses. Anelloviruses (AVs) are among those that distinctly dominate in this environment and are very common in the human population (1–3). The genetic diversity of blood AV isolates is high (4), and coinfections with multiple genera are very common (1, 5). AVs were first discovered in a patient with hepatitis-like symptoms of an unknown etiology (6), but there is currently no convincing evidence for AV pathogenicity (4). AVs are transmitted via transfusions (7, 8); however, they are also present in people who never underwent this medical procedure (1, 9).

Higher levels of AVs are usually found in immunocompromised people, for example, organ transplant patients (10, 11), HIV-1-infected individuals (12), or pregnant women (13). Even though AVs were proposed as a biomarker of immune status (10, 14, 15), the direct relationship between viral load and host immunity and the nature of infection (chronic or acute) remain controversial (4). The anellovirus virome (“anellome”) of an individual may generally be stable over time, which indicates a chronic infection and the host’s tolerance of the anellome. It may even be that once individuals become infected with AVs during early childhood (16, 17), they carry the same variants throughout life. An alternative hypothesis is that an anellome is continuously changing due to clearance of strains and reinfections, which suggests an acute infection under continuous restriction by the host. In order to tackle this dilemma, it is crucial to characterize the healthy human anellome and estimate its stability over time; this may be the first step toward understanding the role of AVs in health and disease. Here, we studied the blood anellomes of 2 healthy people to determine the long-term dynamics of AV infections.

## RESULTS

### General dynamics of anellovirus infection.

Serum samples from 2 male subjects were collected roughly every 6 months. The lengths of the sampling periods were 34.4 and 32.2 years for subjects 1 and 2, respectively. Gaps in sampling occurred between approximately years 11 and 17 for subject 1 and years 9 and 15 for subject 2 due to the suspension of the study. The study covered a continuous follow-up of almost 54 person-years.

**FIG 1:**
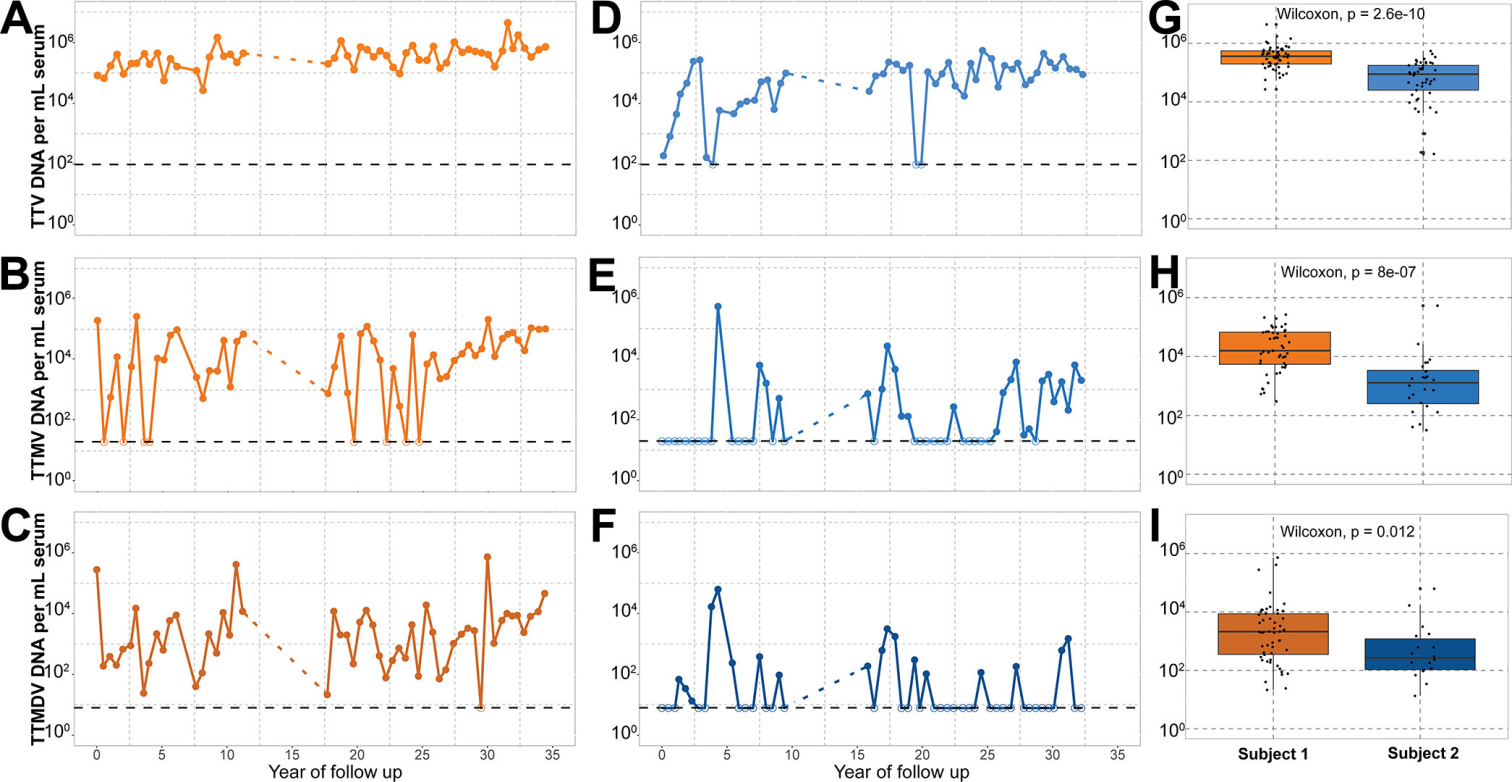
Prevalence of anelloviruses over time. DNA concentrations were measured by qPCRs targeting human-infecting anellovirus genera. (A to F) qPCRs detecting *Alphatorquevirus* (A), *Betatorquevirus* (B), and *Gammatorquevirus* (C) in subject 1 samples and *Alphatorquevirus* (D), *Betatorquevirus* (E), and *Gammatorquevirus* (F) in subject 2 samples. The estimated detection limit of each assay is shown as a black horizontal dashed line, and the samples negative in the assay are depicted as open circles. (G to I) Box plots showing summarized *Alphatorquevirus* (G), *Betatorquevirus* (H), and *Gammatorquevirus* (I) DNA copy numbers in both subjects. The horizontal bar within the box plots depicts the median concentration. The *P* values are shown at the top of each box plot.

**FIG 2:**
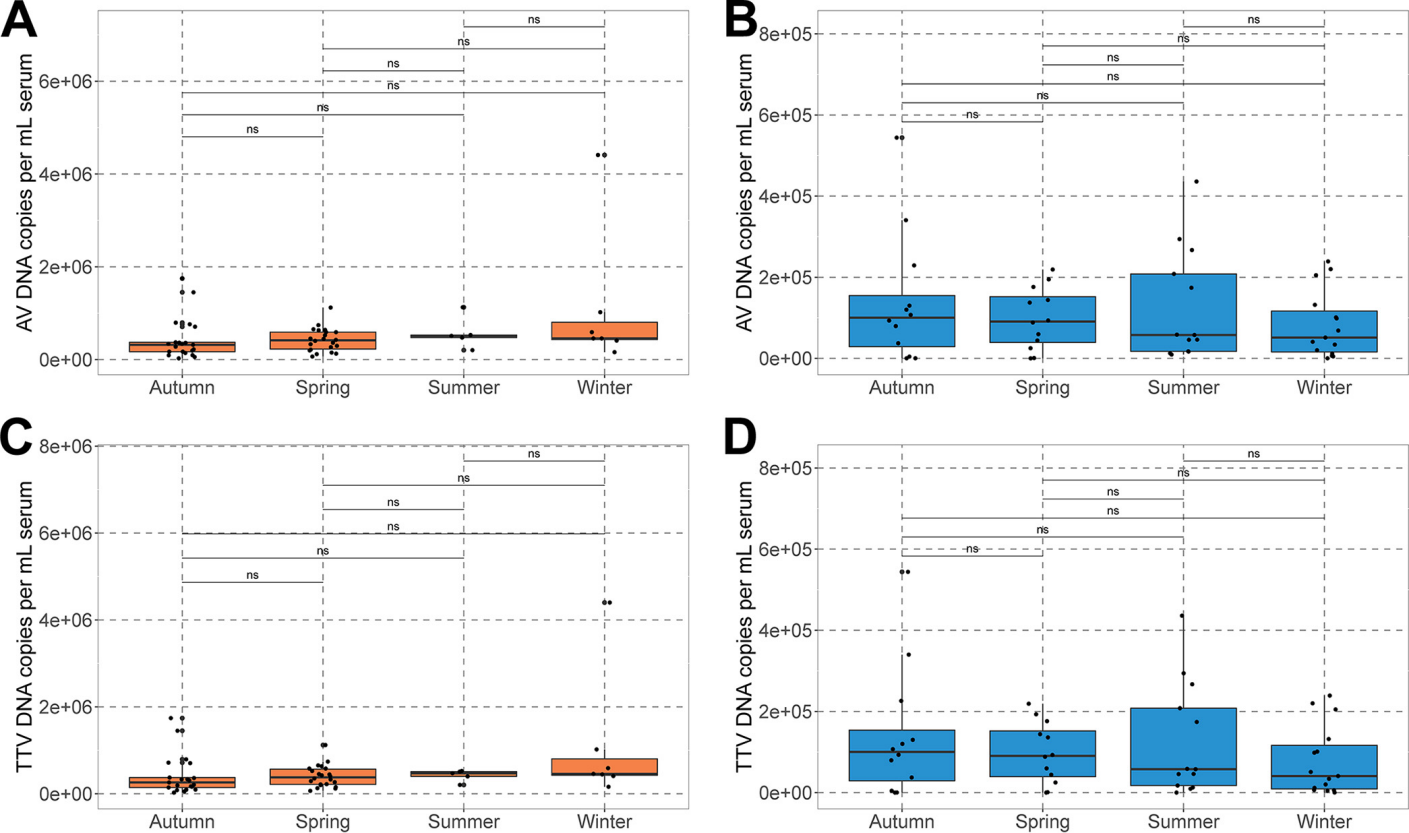
Seasonality of anellovirus infection. (A and B) Subject 1 (A) and subject 2 (B) total AV copy numbers were calculated by summing up the results of all three genus-specific qPCRs. (C and D) Copy numbers of TTV in subject 1 (C) and subject 2 (D). ns, not significant.

In order to assess the presence of AVs in the subjects, nucleic acid (NA) samples were subjected to three genus-specific quantitative PCRs (qPCRs) detecting *Alphatorquevirus* (torque teno virus [TTV]), *Betatorquevirus* (torque teno minivirus [TTMV]), and *Gammatorquevirus* (torque teno midivirus [TTMDV]) (Fig. 1). A list of primers used in the assays is presented in Table S1 in the supplemental material. All samples of subject 1 were positive for AV DNA, while for subject 2, AV DNA was detected in all but three samples (Fig. 1). Both subjects were positive for *Alphatorquevirus* in most of the samples (Fig. 1A and D), while *Betatorquevirus* DNA was less frequently detected (Fig. 1B and E). *Gammatorquevirus* DNA was not detected in the majority of subject 2 samples, while among subject 1 samples, only one time point was negative in the assay (Fig. 1C and F). The median concentrations of *Alphatorquevirus* and *Betatorquevirus* DNA in subject 1 were significantly higher than those in subject 2 (*Alphatorquevirus*, *P* = 2.6 · 10^−10^; *Betatorquevirus*, *P* = 8.0 · 10^−7^ [by a Wilcoxon test]) (Fig. 1G and H), but the difference was not significant for *Gammatorquevirus* (Fig. 1I). We observed moderate increases in *Alphatorquevirus* concentrations over time for both subjects (ρ = ~0.5; *P* = 2.11 · 10^−5^ and 4.52 · 10^−5^ for subjects 1 and 2, respectively) and in *Betatorquevirus* concentrations for subject 2 (ρ = ~0.4; *P* = 0.0019) (Table S4). We also checked whether there was a yearly pattern that might suggest seasonality; however, the AV DNA concentration did not significantly differ across seasons (Fig. 2).

### Anellovirus sequencing output.

Rolling-circle (RC) amplification (RCA) substantially enriches AV DNA (Fig. S2), allowing the construction of high-quality Illumina libraries with over 10^6^ reads per sample on average (Fig. S3A and Table S2). We obtained a total of 20,236,174 AV reads (approximately 14% of the total reads) yet significantly more AV reads for subject 1 samples than for subject 2 (averages of 2.8 · 10^5^ and 9.3 · 10^4^ AV reads, respectively; *P* = 4.7 · 10^−7^ [by a Wilcoxon test]) (Fig. S3B and Table S3), mirroring the virus concentrations in the samples.

The *de novo* genome assembly of paired reads resulted in 5,755 and 15,213 scaffolds (>1,000 bp) for subject 1 and subject 2, respectively (Fig. S3C and Table S2). The scaffolds were subjected to a virus discovery pipeline, which resulted altogether in 649 AV scaffolds for subject 1 and 105 AV scaffolds for subject 2 (Fig. S3D and Table S2). After curation to remove nearly identical sequences (using a 95% identity with a 95% coverage threshold, performed separately for each subject), the final catalogue included 53 AV lineages for subject 1 and 11 lineages for subject 2.

### Diverse and personal anellome.

The Cenote Taker 2 virus discovery pipeline annotates scaffolds based on various protein databases (18), and we used the annotations to catalogue each unique AV lineage into known AV species. We identified a total of 25 AV species in the subjects (Fig. S3E). There was a visible dominance of *Alphatorquevirus* over the other *Anelloviridae* genera. The majority of reads (more than 6 million) in subject 1 aligned to lineages identified as TTV24 species. The dominating species in subject 2 was TTV6. The most prevalent *Betatorquevirus* species in subject 1 was TTMV12, and in subject 2, it was TTMV10. Although there were a few time points that were PCR positive for *Gammatorquevirus* DNA (Fig. 1F), no *Gammatorquevirus* lineages were identified in subject 2 by sequencing. However, an inspection of scaffolds discarded from subject 2 because of insufficient length revealed one scaffold that had a hit to *Gammatorquevirus* (species TTMDV10) (data not shown). In subject 1, there were some more *Gammatorquevirus* lineages found, with TTMDV15 being the most prevalent species. Furthermore, we identified some potentially novel AV species (Fig. S3E and Table S3).

**FIG 3:**
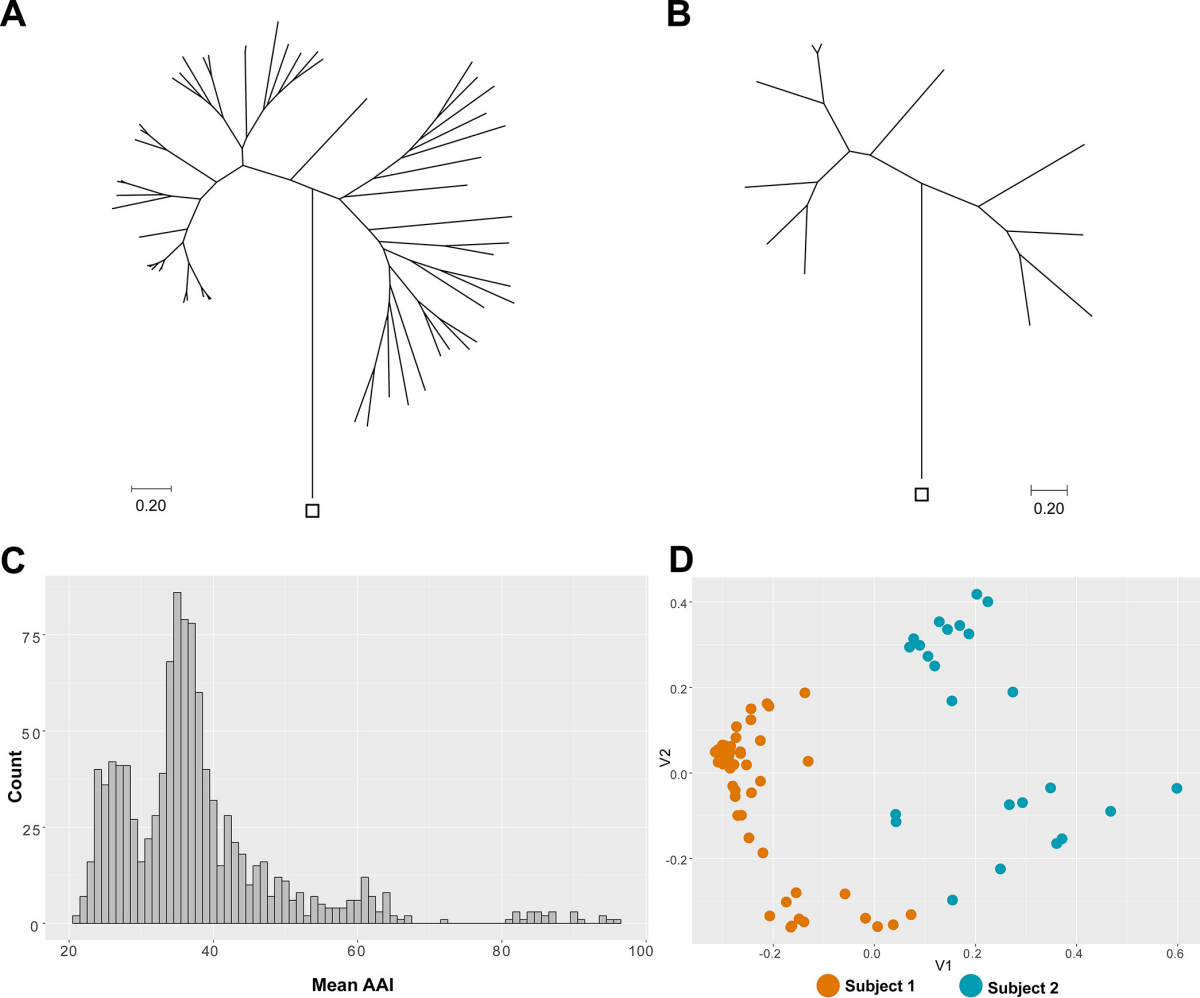
Highly diverse and personal anellome. (A and B) Phylogenetic relationships of 53 subject 1 (A) and 11 subject 2 (B) anellovirus lineages. The trees were constructed using the maximum likelihood method and are based on amino acid alignments of ORF1 proteins. The outgroup (rodent anellovirus [GenBank accession number NC_040687]) is indicated with a square. (C) Pairwise amino acid identity (AAI) comparison between anellovirus lineages derived from both subjects. All ORF1 sequences were compared against each other. (D) Principal-coordinate analysis (PCoA) ordination of the viral community matrix based on the unweighted UniFrac divergence metric. The color code of the subjects is given at the bottom.

To visualize the overall diversity of AVs in each subject, we constructed maximum likelihood phylogenetic trees based on the amino acid sequence of the AV capsid protein, referred to as open reading frame 1 (ORF1) (Fig. 3A and B; Fig. S4). We confirmed the protein annotations obtained by Cenote Taker 2 since lineages sequenced from our subjects resolved alongside the proper reference strains in a global tree (Table S3 and Fig. S4). The overall mean genetic distances were similar (0.65 and 0.70 for subject 1 and subject 2, respectively).

We found that only 2 out of 11 subject 2 lineages showed high identity (>95% on the nucleotide level) across the whole genome with lineages from subject 1. In addition, we compared the pairwise amino acid identities of ORF1 sequences from both subjects (Fig. 3C; raw data are presented in Table S5). The majority of pairs showed <40% identity for the ORF1 protein sequence, which suggests that sharing of lineages between people is very sporadic, and it points toward the hypothesis that each person carries a personal anellome.

### Persistence of lineages over time.

**FIG 4:**
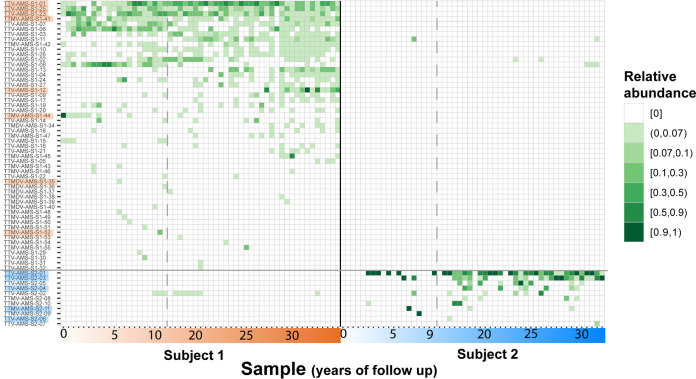
Anellovirus diversity and prevalence over time. A heatmap shows the relative abundances of various anellovirus lineages in subject 1 and subject 2 over time. The color strength represents the relative abundance of a lineage per time point. The approximate years of follow-up are shown on the *x* axis. The lineages were arranged on the *y* axis based on the number of positive subject time points. The lineages highlighted with orange or blue were also tested by specific qPCR. The dashed lines denote sampling gaps.

A large number (64) of AV lineages were found in the subjects, but their temporal dynamics were unknown. We mapped sequencing reads from each sample to the curated lineage database. Based on the numbers of mapped reads, we calculated the proportion of reads aligning to each lineage for each time point. First, we wanted to assess whether the individual time points of the subjects showed differences in their phylogenetic profiles. We performed unweighted UniFrac analysis, which uses phylogenetic information and the presence or absence of each detected lineage in each sample to compare all the samples within a data set. The principal-coordinate analysis (PCoA) of the unweighted UniFrac comparison (Fig. 3D) displayed a clear separation of subject 1 samples from subject 2 samples, which indicates that the phylogenetic profiles of the samples cluster by subject and points toward a personal and stable anellome. Moreover, the normalized Shannon index of subject 1 was significantly higher than that of subject 2 (*P* = 1.2 · 10^−9^ by a Wilcoxon rank sum test) (Fig. S5A), and the median number of lineages per time point reached 11 for subject 1, versus 2 for subject 2 (Fig. S5B). The large anellome of subject 1 remained so over time, and the anellome of subject 2 was stably small over more than 30 years (Fig. 4; Fig. S5B).

A total of 44 lineages from either subject (35 of subject 1 and 9 of subject 2) were detected more than once during follow-up. In subject 1, 8 lineages were detected in over half of the samples, with 2 being present at 93% of the time points (Fig. S6). In subject 2, the most persistent lineage was found in 69% of the samples (Fig. S6). Of the 44 AV lineages, 4 from subject 1 were detected exclusively in the first 10 years of follow-up, and 7 lineages were detected only after 10 years had passed (Fig. 4). In subject 2, there were 4 lineages that first appeared after 10 years. Importantly, however, most lineages (24 of 35 and 5 of 9 for subject 1 and subject 2, respectively) were detected across the whole follow-up period. Therefore, losses of existing lineages and new introductions were rare in both subjects.

Twenty lineages were detected only once: 18 lineages of subject 1, belonging mostly to *Betatorquevirus* (8 lineages) and *Gammatorquevirus* (6 lineages), and 2 *Alphatorquevirus* lineages of subject 2 (Fig. 4; Fig. S6). These members of the “transient anellome” represented 34% and 18% of all lineages of subjects 1 and 2, respectively.

As mentioned above, 2 lineages of subject 1 (TTV-AMS-S1-02 and TTV-AMS-S1-11) showed high identity with lineages from subject 2 (TTV-AMS-S2-02 and TTV-AMS-S2-03, respectively) (Fig. S4). It is therefore not surprising that some subject 2 reads aligned with lineages of subject 1 and vice versa (Fig. 4).

**FIG 5:**
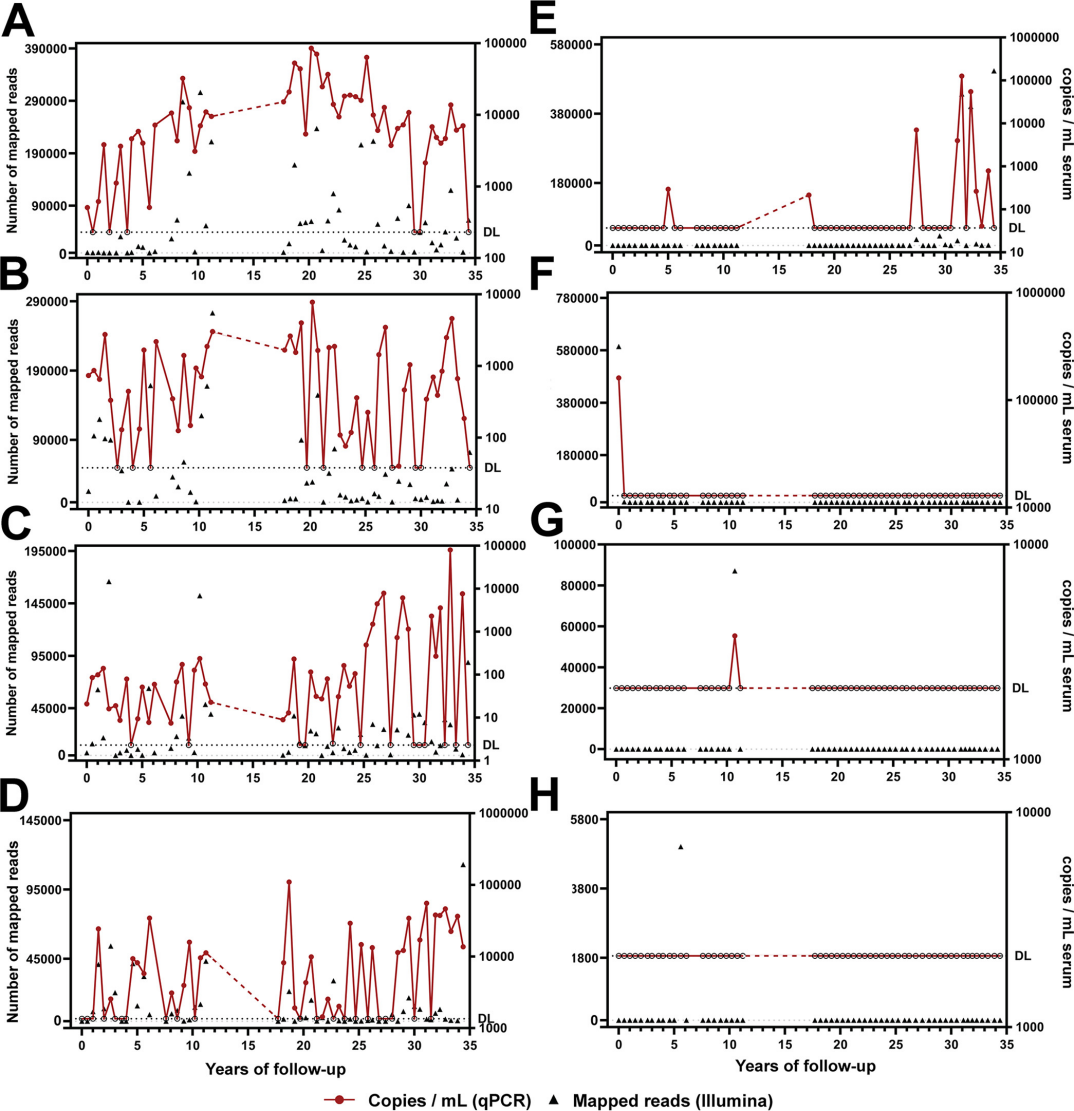
Persistence of selected anellovirus lineages over time in subject 1. Eight lineages were selected for specific qPCR analysis. The graphs show the results of mapping of the Illumina reads to the selected lineage (left *y* axis) and the copies per milliliter measured using lineage-specific qPCR (right *y* axis). The analysis was performed for the following lineages: TTV-AMS-S1-01 (A), TTV-AMS-S1-23 (B), TTV-AMS-S1-25 (C), TTMV-AMS-S1-41 (D), TTV-AMS-S1-12 (E), TTMV-AMS-S1-44 (F), TTMV-AMS-S1-52 (G), and TTMDV-AMS-S1-35 (H). One out of two performed qPCR runs is plotted here. The dashed horizontal line marked with “DL” represents the estimated limit of detection of the qPCR assay. The samples that were negative by qPCR are depicted with an open circle.

To ascertain the long-term or short-term persistence of lineages, qPCRs targeting specific AV lineages were performed. The primer design was performed cautiously so that the primers and probes were targeting the lineage of interest exclusively (Table S1). We confirmed the long-term persistence of 4 lineages in subject 1 (Fig. 5A to D; Fig. S7A to D). Furthermore, we confirmed the short-term persistence of subject 1 lineage TTV-AMS-S1-12 (Fig. 5E; Fig. S7E). NGS showed its presence only at the end of the follow-up; however, we also observed a qPCR-positive sample at year 5 (Fig. 4 and Fig. 5E). This finding suggests that this lineage or a close relative was present in the subject’s blood at the beginning of follow-up. Another lineage was detected by NGS only at the few first time points, with the very first time point containing almost 600,000 mapped reads (TTMV-AMS-S1-44) (Fig. 5F). qPCR confirmed this finding, with more than 10^5^ copies per mL in that sample, and the other samples were negative (Fig. 5F). Two lineages were detected at a single time point (TTMV-AMS-S1-52 and TTMDV-AMS-S1-35) (Fig. 4). This was confirmed by TTMV-AMS-S1-52 DNA-specific qPCR, yet all samples were negative by TTMDV-AMS-S1-35 DNA-specific qPCR (Fig. 5G and H).

**FIG 6:**
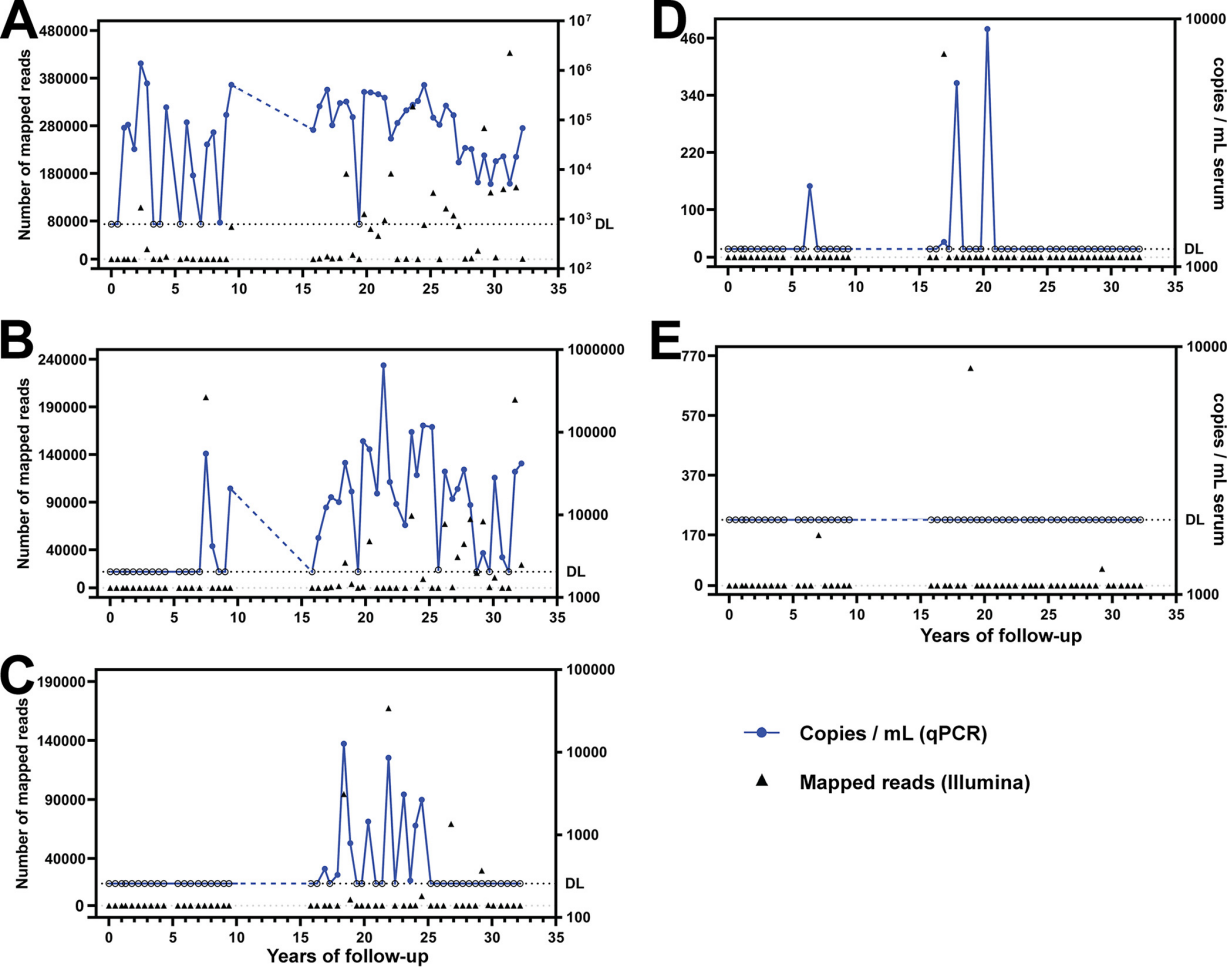
Persistence of selected anellovirus lineages over years in subject 2. Five lineages were selected for specific qPCR analysis. The graphs show the results of mapping of the Illumina reads to the selected lineage (left *y* axis) and the copies per milliliter measured using lineage-specific qPCR (right *y* axis). The analysis was performed for the following lineages: TTV-AMS-S2-01 (A), TTV-AMS-S2-03 (B), TTV-AMS-S2-04 (C), TTV-AMS-S2-06 (D), and TTMV-AMS-S2-11 (E). One out of two performed qPCR runs is plotted here. The dashed horizontal line marked with “DL” represents the estimated limit of detection of the qPCR assay. The samples that were negative by qPCR are depicted with an open circle.

In the case of subject 2, we PCR confirmed the persistence of two potentially chronic lineages (Fig. 6A and B; Fig. S7F and G) and the transient or scattered presence of two other lineages (Fig. 6C and D; Fig. S7H). The presence of lineage TTMV-AMS-S2-11 could not be PCR confirmed (all PCRs were negative) (Fig. 6E). This lack of detection may have been caused by a very low copy number of certain lineages in blood.

## DISCUSSION

In this study, we explored the blood anellomes of two healthy individuals for over 30 years. Much remains to be learned about the blood virome, an environment in which AVs are highly abundant (1). Recent studies using viral metagenomics included transplant patients (2, 10, 19) or blood transfusion recipients (8). Our study involved healthy people who did not receive a blood transfusion or an organ transplant; therefore, we had a unique opportunity to look at the stability of the natural anellome. At study entry, subjects 1 and 2 were 41 and 35 years of age, respectively, whereas the last blood samples were collected when they were aged 75 and 68 years, respectively. We observed a moderate but significant increase in *Alphatorquevirus* concentrations over time for both subjects. Studies of solid-organ transplant recipients have shown a direct relationship between immune suppression and the quantity of anelloviruses in the blood (10, 20, 21). The rise in the viral load that we observed in time is plausibly related to the declining quality of the immune system caused by aging. It was reported previously that *Alphatorquevirus* DNA loads are significantly higher in the elderly than in young people (22).

A dominance of *Alphatorquevirus* over other AV genera was observed throughout the >30 years of follow-up, and the concentration was higher in subject 1 than in subject 2. The median concentration of *Betatorquevirus* was also significantly higher for subject 1. Lineages in both subjects were widely found across the phylogenetic tree, with the exception of the *Gammatorquevirus* genus, which was not found in subject 2. Our findings are in line with the results of Arze and colleagues, who also showed that smaller anellomes can capture the breadth of AV taxonomy (8). A less rich anellome was associated with decreased concentrations of *Alphatorquevirus* (see Fig. S5C to E in the supplemental material). However, some samples of subject 2 showed high concentrations (10^5^ *Alphatorquevirus* DNA copies per mL) and still contained a maximum of 5 AV lineages, and we therefore consider the low richness of subject 2’s anellome stable.

Several AV lineages persisted in the subjects. These chronic lineages may be considered the “core anellome” of an individual. We are the first to report an unceasing persistence of AV lineages for such an extended period as 30 years. Studies quantifying specific AV lineages in time are scarce and cover shorter time periods but nevertheless are consistent with our findings. Arze et al. showed the persistence of AV lineages in 3 people for almost 9 months (8). In a study by Jazaeri Farsani et al., a *Betatorquevirus* genotype was present for more than 42 months in the blood of an HIV-1-infected individual (23), and Bédarida et al. also showed the long-term (16 years) persistence of an AV lineage in human plasma; however, the latter study was based on only two time points (24). On the other hand, Segura-Wang and colleagues observed strong fluctuations in the anellome compositions in plasma and bronchoalveolar lavage (BAL) fluid samples collected from lung transplant recipients for around a year after the transplant (19). These changes in the anellome in transplant recipients may have been caused by the introduction of the viruses from the donor and the administration of immunosuppressive drugs, which are known to affect AV abundance (10, 19). Of note, even though the observed changes were substantial, a few AV lineages were still detected at more than one time point (19). Besides the core anellome, we observed 15 lineages that were detected only in a restricted period of the follow-up and 20 lineages that were detected at a single time point. Although we cannot exclude that the lineages persist at a concentration below the detection limit of our assay, it could be that these lineages represent newly introduced AV lineages that are subsequently cleared. In that line of thinking, we postulate that the core personal anellome contains lineages that are tolerated and carried throughout life. Future longitudinal serological studies will be needed to examine the immune responses elicited by the core and transient AV lineages and establish the mechanism of the host’s regulation of the anellome. Humans are infected by AV in early childhood (16, 17), but the source of infection and anellome dynamics in early life are still unknown. It would thus be of interest to determine when the core anellome is established and how it is shaped from the moment of birth until adulthood.

AV DNA is frequently detected in a variety of human organs and tissues (reviewed in detail by Spandole et al. [25]). It is therefore possible that by sampling only the blood, we missed a number of AV lineages that were present in other body compartments. A study by Segura-Wang and colleagues compared the anellomes of paired plasma and BAL fluid samples (19). Most of the AVs found in BAL fluid were also detected in paired plasma samples, and AV richness was generally higher in plasma than in BAL fluid. This finding suggests that sampling only the blood compartment would have been sufficient to obtain a complete anellome landscape of the tested subjects (19). Still, future studies of AV diversity and long-term dynamics in various body compartments are needed to assess whether different organs and tissues harbor distinct anellome compositions and dynamics.

In summary, our data show the dynamics of the blood anellomes of two healthy subjects and demonstrate that anelloviruses may persist in the host for over 30 years. The introductions of new lineages and the clearance of older lineages are rare, and the core, chronic anellome remains stable and personal for many years.

## MATERIALS AND METHODS

### Ethics approval and consent to participate.

Longitudinally collected sera were obtained through the Amsterdam Cohort Studies on HIV Infection and AIDS. This study was approved by the Medical Ethics Committee of the Amsterdam University Medical Center (location AMC) of the University of Amsterdam, The Netherlands (MEC 07/182). Participation in that study is voluntary and without incentive. Written informed consent was obtained from each participant at enrollment.

### Clinical samples.

Fifty-five and 52 serum samples were collected longitudinally from subject 1 and subject 2, respectively. Subject 1 was 41 years old and subject 2 was 35 years old at the start of follow-up. The samples were collected every few months for 413 months (subject 1) and 386 months (subject 2). The samples were collected asynchronously every 3 to 18 months (median of 6 months for both subjects). In the periods of the 132nd to 208th months (3 December 1996 to 10 April 2003) for subject 1 and the 111th to 187th months (16 December 1996 to 7 April 2003) for subject 2, the study was suspended, which resulted in the absence of samples in these periods. The total follow-up period was 54 person-years. Both subjects were volunteer participants in the Amsterdam Cohort Studies on HIV Infection and AIDS (26), which consist of men who have sex with men who live in the Amsterdam area in The Netherlands. The chosen participants were seronegative for HIV-1 throughout the sampling period. Subject 2 had a hepatitis B virus (HBV) infection in 1998. No blood disease, autoimmune disease, cancer, or neurodegenerative disease was described during the follow-up period for the subjects. The serum samples were stored in −80°C freezers after collection.

### Nucleic acid isolation.

After thawing at room temperature, the serum samples were centrifuged for 10 min at 5,000 × *g*. The supernatant was treated with Turbo DNase (Invitrogen), and afterward, the nucleic acids were extracted using the Boom isolation method (27). The nucleic acids were stored at −80°C until further use.

### Genus-specific qPCRs.

The primers and probe detecting *Alphatorquevirus* were designed previously (28), and the primers detecting *Betatorquevirus* and *Gammatorquevirus* were designed especially for this study (see Table S1 in the supplemental material). Dilutions of a positive-control plasmid were used to construct the standard curve. The qPCR mixture consisted of 2.5 μL isolated nucleic acids (before or after rolling-circle amplification [RCA]), 6.25 μL 2× Qiagen RotorGene probe master mix (catalogue number 204374; Qiagen), 0.25 μL probe, 0.5 μL forward and 0.5 μL reverse primers (all 10 μM), and 2.5 μL H_2_O in each sample. The reaction was performed on a RotorGene machine (Qiagen) as follows: 95°C for 3 min, followed by 40 cycles of 95°C for 3 s and 60°C for 10 s and a final elongation step at 72°C for 3 min.

### Rolling-circle amplification.

RCA was performed on the extracted nucleic acids to increase the yields of circular DNAs. Phi29 polymerase (Thermo Scientific) was mixed with the supplied reaction buffer, deoxynucleoside triphosphates (dNTPs) (final concentration, 10 μM; Illustra), exonuclease (exo)-resistant random primers (Thermo Scientific), and dithiothreitol (DTT) (final concentration, 1 μM; Invitrogen). The reaction mixture was incubated at 30°C for 4 h, followed by an enzyme inactivation step for 10 min at 65°C. The RC-amplified samples were stored at −20°C until further use.

### Illumina NGS library preparation.

Illumina library preparation was carried out using a method developed in-house, based on the New England BioLabs (NEB) Next protocol, with the following modifications. Between each enzymatic reaction, AMPure XP bead cleanup was performed (without size selection, unless stated otherwise). The RC-amplified samples were fragmented using Fragmentase enzyme at 37°C for 25 min. The ends of fragmented DNAs were repaired using polymerase I, large (Klenow) fragment (NEB), in combination with NEB2 10× buffer (NEB) and dNTPs (final concentration, 500 μM each) at 37°C for 30 min. A-tailing was performed with 3′-5′ exo^−^ polymerase I, large (Klenow) fragment (NEB); NEB2 buffer; and dATPs (final concentration, 200 μM; NEB) at 37°C for 30 min. NEB Next adaptors were mixed with T4 ligase (Invitrogen) and T4 buffer provided by the supplier. The ligation reaction mixture was incubated overnight at 16°C. AMPure XP bead size selection was performed before the enrichment of adaptor-ligated DNA. A ratio of 1 (ligation product) to 1.7 (AMPure beads) was used. The samples were eluted in 20 μL of nuclease-free water, and 10 μL of the elution mixture was used for adaptor enrichment PCR. The PCR master mix contained Q5 Hot Start master mix (NEB), NEB Next universal primer (final concentration of 0.5 μM), NEB Next index primer (final concentration of 0.5 μM), and USER enzyme (NEB). Each sample had an assigned unique index primer. Cycling was performed as follows: 37°C for 15 min and 98°C for 30 s, followed by 12 cycles of 98°C for 10 s and 65°C for 75 s. There was a final extension step at 65°C for 5 min. After the PCR, two rounds of AMPure XP bead size selection were performed to remove fragments of undesired lengths. After elution in 20 μL of Baker water, the concentration of each sample was measured using a Qubit high-sensitivity assay (Invitrogen), and the samples were pooled at equal concentrations and run on the Illumina MiSeq platform. The samples from different subjects were processed and run separately.

### Processing of Illumina sequencing data and viral classification.

Raw sequence reads in .fastq format were first analyzed for their quality using FastQC (29) (version 0.11.9) and visualized in MultiQC (30) (version 1.9). The adaptors and low-quality reads were trimmed using Trimmomatic (31) (version 0.39). The SPAdes genome assembler (32) (version 3.14.0) was used in the standard mode, with –careful and –only-assembly settings. The assembled scaffolds were subjected to viral genome discovery using the Cenote Taker 2 pipeline (18). The scaffolds that were identified as potential AVs were additionally checked using BLASTn alignment to the NCBI nucleotide database (33) and, if necessary, corrected manually. The obtained full-length or nearly full-length (>2-kb) genomes were transferred into Codon Code Aligner (version 8.0.2) and (per-subject) assembled using a 95% identity threshold. The assemblies were visually inspected, and one representative genome was selected from each assembly. Afterward, the selected lineages were additionally checked using ORFfinder (https://www.ncbi.nlm.nih.gov/orffinder/) for the presence of the ORF1 gene. If the ORF1 gene could not be found, the sequences were discarded from further analysis. The selected sequences were also visually inspected in Codon Code for their read coverage across the genome. Poorly covered or chimeric regions were manually trimmed. In cases where doubtful regions were in the middle of the genome, they were confirmed by Sanger sequencing. The quality-checked AV lineages were uploaded to GenBank (Table S3), and together, these lineages formed the curated AV lineage database.

### Prevalence of anelloviruses over time.

To determine the prevalence of the AVs over time, sequencing reads from each time point were aligned to the curated AV lineage database using bowtie2 (34) (in –very-sensitive, end-to-end mode) and a reads-to-genomes table was generated using SAMtools (35). The read coverage across the genome was calculated using the BBMap pileup function. A read coverage of ≥75% of the genome length (covered at least once) was considered a hit. The Lisa high-performance computing cluster (Surfsara [https://www.surf.nl/]) was used for the majority of the data analysis work.

### Phylogenetic analysis of ORF1 proteins.

ORF1 protein sequences were extracted from the anellovirus genomes using emboss getorf (http://emboss.bioinformatics.nl/cgi-bin/emboss/getorf). The amino acid sequences were aligned using MAFFT (36) (E-INS algorithm), and the maximum likelihood phylogenetic trees were constructed and the overall mean distance was calculated using MEGAX software (37) (50 bootstraps).

### Lineage-specific qPCR.

Specific qPCRs targeting selected anellovirus lineages were performed in order to confirm the data obtained by Illumina NGS. Each primer-and-probe set was specific for a selected anellovirus lineage (Table S1). The primers were designed using Codon Code Aligner. In order to prepare the positive-control plasmid, PCR was performed as follows: 95°C for 5 min, followed by 35 or 25 cycles of 95°C for 30 s, 55°C for 30 s, and 72°C for 1 min each and a final elongation step at 72°C for 10 min. A nested setting was required for the majority of the viruses; only two lineages, TTMV-AMS-S1-41 and TTV-AMS-S1-23, did not require the nested reaction. Both the first PCR (35 cycles) and nested PCR (25 cycles) were performed using master mix containing 12.5 μL of DreamTaq green PCR master mix (catalogue number K1081; Thermo Fisher Scientific, Waltham, MA, USA), 0.25 μL of forward and 0.25 μL of reverse primers (both 20 μM), 9.5 μL of H_2_O, and 2.5 μL of the template (serum NAs or the 1:10-diluted product of the previous PCR). The (nested PCR) products of each of the specific reactions were cloned into the pCR 2.1 vector using a TA cloning kit (Invitrogen) and, after serial dilution, were used to construct a standard curve for the qPCR analysis. Only the nested primers were used in the qPCRs. The qPCRs were performed in the same way as the genus-specific qPCR, but a higher number of intertwined no-template controls (NTCs) was included (2 NTCs every 5 samples), and for lineage-specific qPCR, the reaction consisted of 45 cycles. The runs were analyzed in the same way as the genus-specific qPCRs. Combined graphs of one of the qPCR runs and the number of mapped Illumina reads were constructed using GraphPad Prism software (version 9.1.0).

### Statistical analysis.

Analysis of alpha diversity (Shannon index) was performed in R version 4.1.1 equipped with the Vegan package (v.2.5-7). The Shannon index was normalized based on the highest diversity value of a subject. UniFrac analysis was performed in QIIME 2 (38) version 2021.8.0 using the Lisa high-performance cluster computer (Surfsara [https://www.surf.nl/]). Both the presence of viruses and phylogenetic relationships are considered in UniFrac. We performed unweighted UniFrac analysis, which takes only the presence or absence of a lineage into account. The principal-coordinate analysis graphs were constructed in R equipped with the Vegan package. All box plot, heatmap, linear regression, and bar chart figures were constructed in R using the ggplot2 package. The box represents the standard Tukey type with the interquartile range, and the bar within it represents the median. Statistics were performed in R using the stats package.

### Data availability.

Raw sequence files and metadata for all tested samples have been deposited under NCBI BioProject accession number PRJNA785545. The novel anellovirus genomes were deposited to GenBank genome database under accession numbers OL694779-OL694842. The workflow of the analysis is available on GitHub (https://github.com/joannakaczorowska/Anellome_is_stable).
