# Supplementary material for: Diversity and Long-Term Dynamics of Human Blood Anelloviruses
Source: J Virol. 2022 May 16;96(11):e00109-22. doi: 10.1128/jvi.00109-22 (PMC9175625; doi:10.1128/jvi.00109-22)
Supplement: Supplemental file 1 — Figures S1 to S7. Download jvi.00109-22-s0001.pdf, PDF file, 1.3 MB [file jvi.00109-22-s0001.pdf]

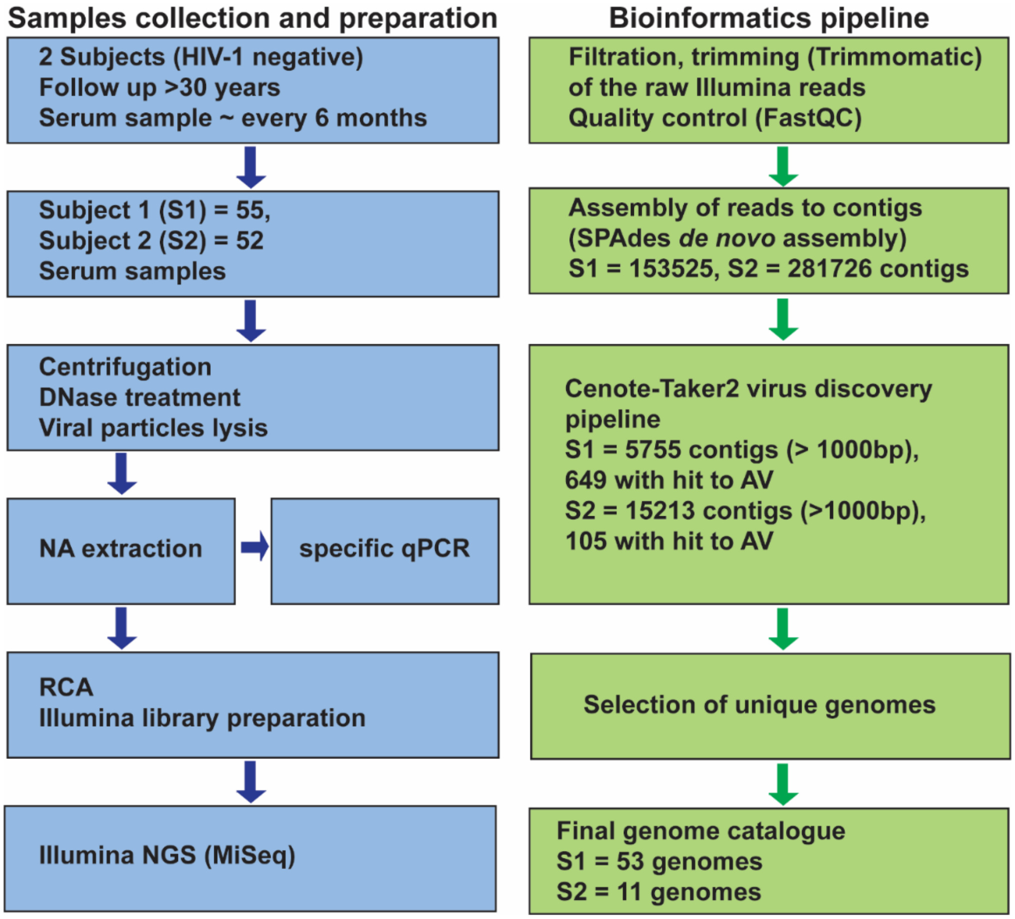

Figure S1. An overview of experimental and bioinformatics methods used to obtain genome catalogues from two tested subjects (S1 – Subject #1, S2 = Subject #2).

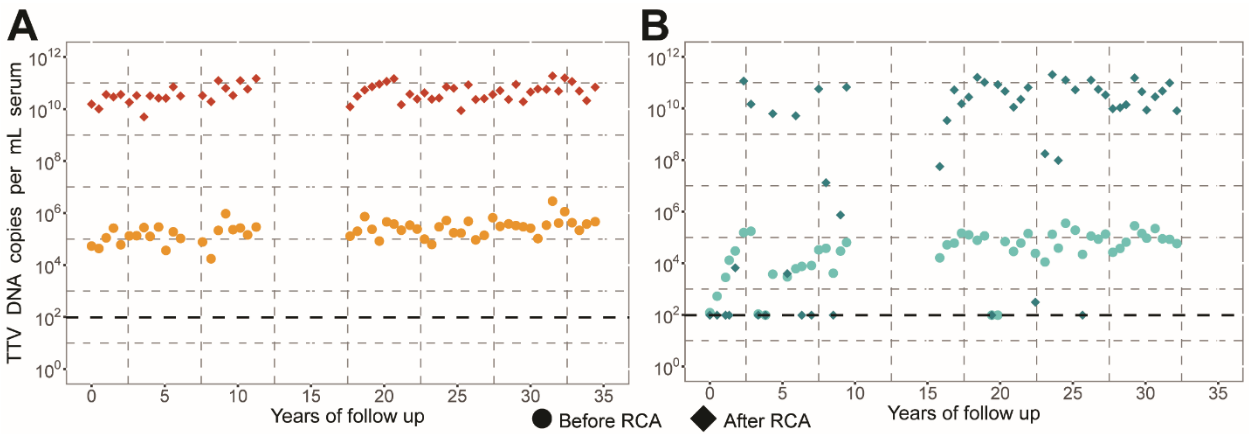

Figure S2. TTV-genus specific qPCR before (circles) and after (diamond shape) rolling circle amplification for subject #1 (A) and subject #2 (B). The estimated detection limit of each assay is shown as a black horizontal dashed line.

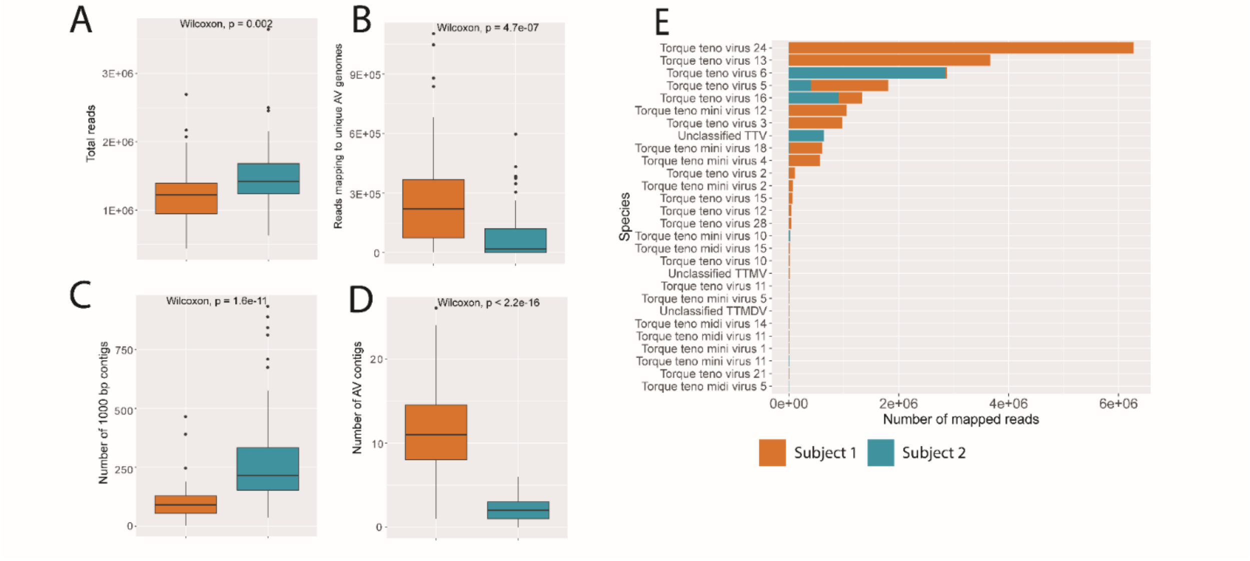

Figure S3. A) Total number of Illumina reads, (B) Illumina reads mapping to unique anellovirus genomes (lineages), (C) total number of > 1000 bp contigs and (D) number of contigs identified as AVs in all timepoints from subject 1 and subject 2. (E) Prevalence of anellovirus species in each subject. The classification is based on the annotations obtained in the Cenote Taker 2 virus discovery pipeline and the counts are the numbers of paired reads mapped to the genome database.

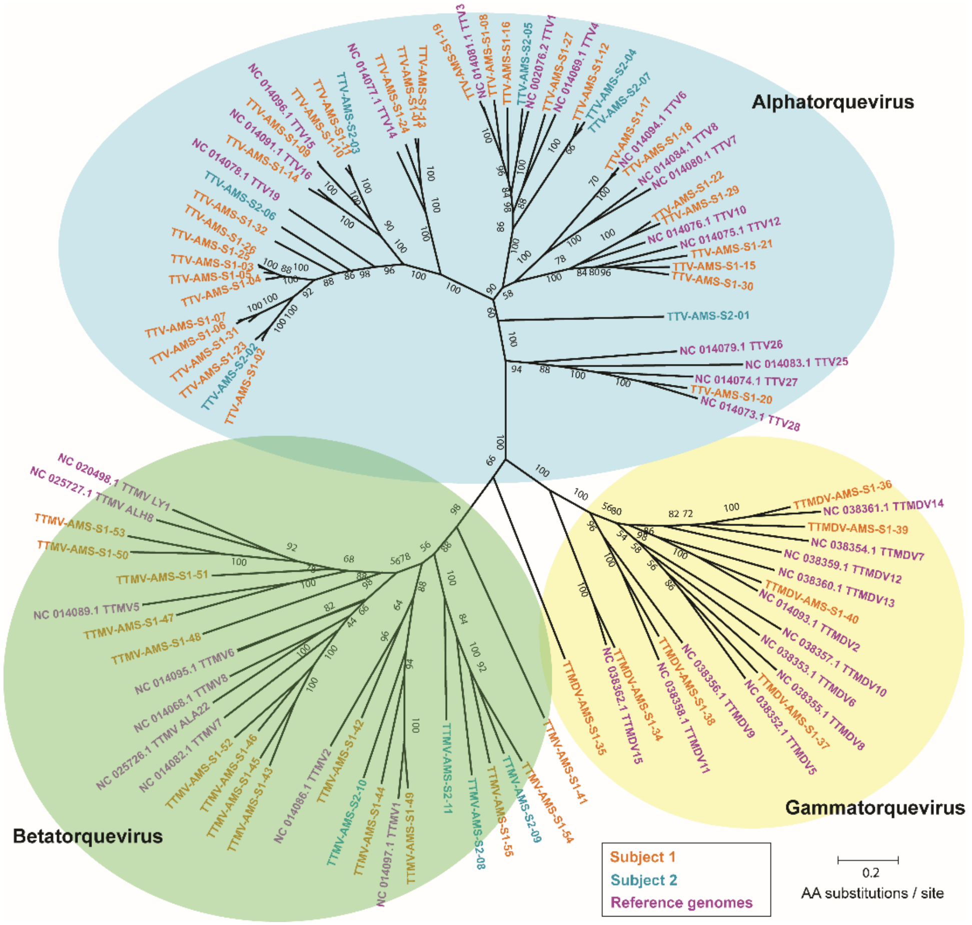

Figure S4. Phylogenetic relationship of the AV genomes obtained from the subject #1 (indicated in orange color) and subject #2 (in blue) and reference strains (in purple). The maximum likelihood tree is based on the ORF1 amino acid alignment. AV genera are indicated with color planes, blue for Alphatorquevirus (TTV), green for Betatorquevirus (TTMV) and yellow for Gammatorquevirus (TTMDV).

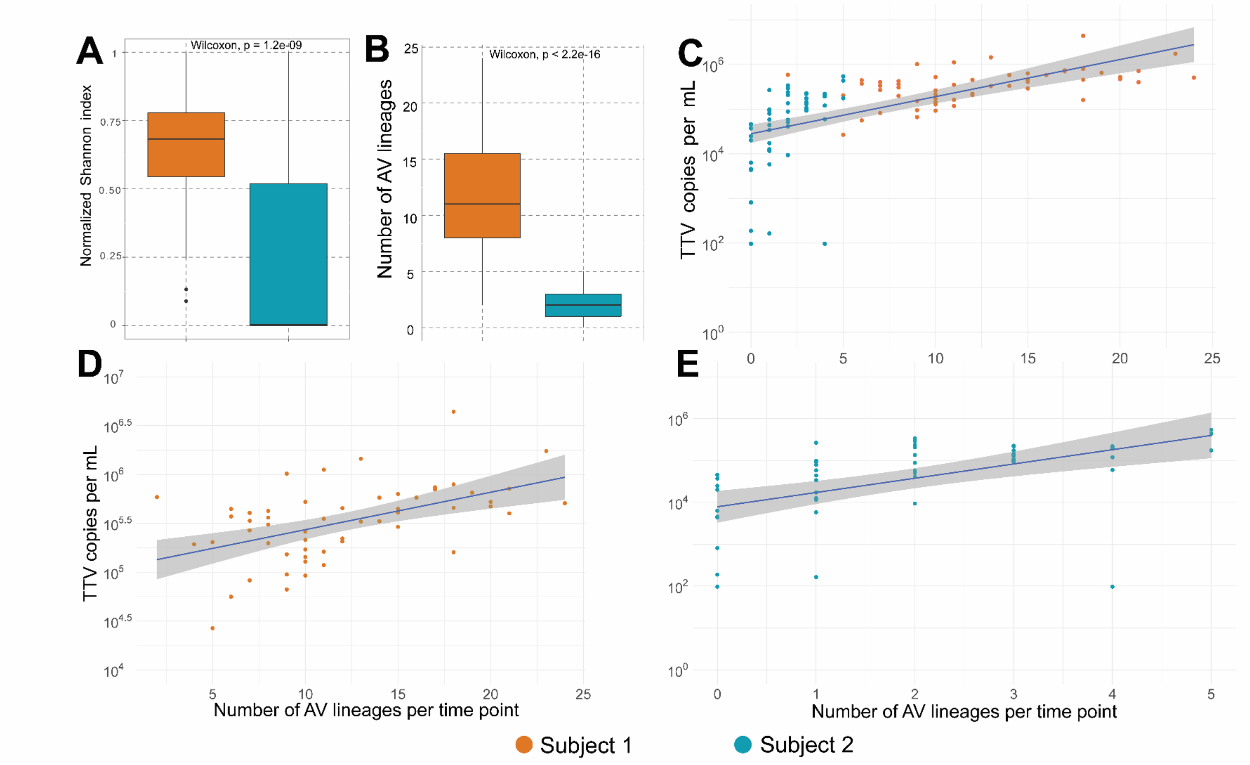

Figure S5. Alpha diversity of the anellome and number of lineages per sample. Boxplot of subject #1 and subject #2 normalized Shannon index (A) and of number of lineages per time point (B). The normalization was performed based on the sample with the highest Shannon index (per person). Correlation of concentration of TTV copies per mL of the serum with the number of lineages detected in each sample: (C) for both subjects, (D) subject #1 only, (E) subject #2 only.

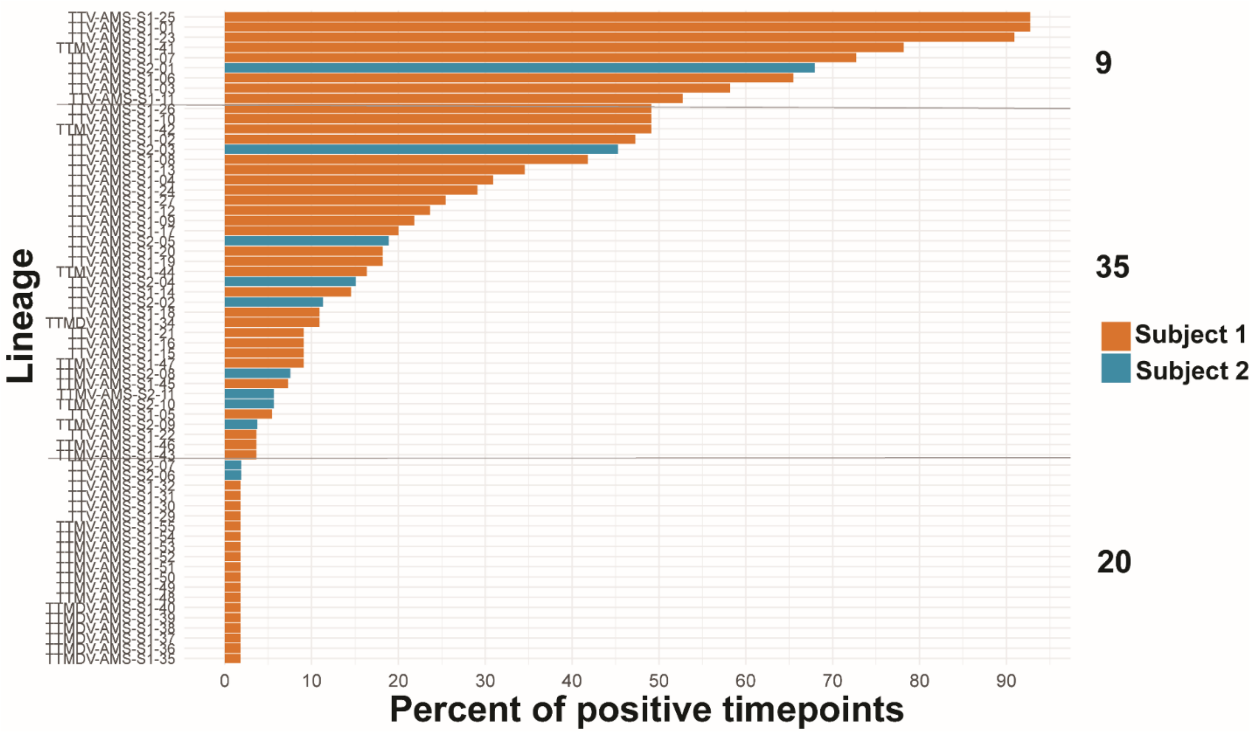

Figure S6. Percent of timepoints positive for anellovirus lineages. Nine lineages were detected in more than 50 % of the time points, 35 in less than 50 % but more than one time point, and 20 lineages were found in only one time point.

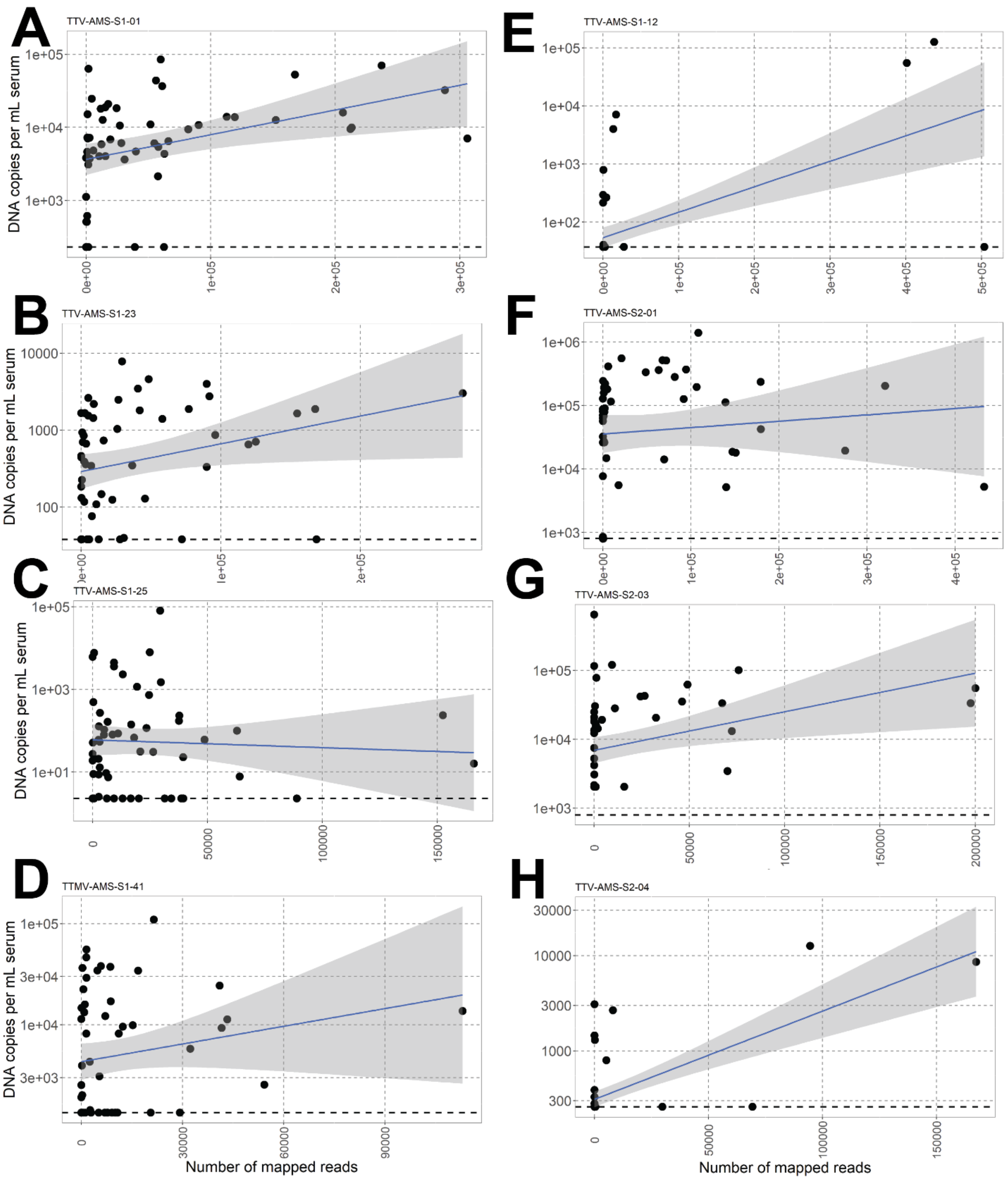

Figure S7. Relationship between the virus load and the number of mapped reads in each timepoint for lineages that were detected in multiple time points. A) TTV-AMS-S1-01, (B) TTV-AMS-S1-23, (C) TTV-AMS-S1-25, (D) TTMV-AMS-S1-41 (E) TTV-AMS-S1-12, (F) TTV-AMS-S2-01, (G)

TTV-AMS-S2-03 and (H) TTV-AMS-S2-04. The dashed horizontal line represents the detection limit of the qPCR.
